# Unusual Experience in the COVID-19 Pandemic: Young Children’s Readjustment from Home to Preschool following School Closures in Different Risk Regions

**DOI:** 10.3390/ijerph192416785

**Published:** 2022-12-14

**Authors:** Xiumin Hong, Sijie Zhao, Qianqian Liu

**Affiliations:** Faculty of Education, Beijing Normal University, No. 19 Xin Jie Kou Wai Street, Hai Dian District, Beijing 100875, China

**Keywords:** young children, preschool readjustment, COVID-19, risk regions

## Abstract

Children’s readjustment to preschool following long-term school closures during the COVID-19 pandemic merits special attention. This study examined children’s preschool readjustment using a survey of 1008 teachers in a high-risk region and 1399 teachers in a fluctuating-risk region of China. Results found are as follows. (1) children’s preschool readjustment was at a medium level after the long-term school closures. However, children’s preschool readjustment scores in the fluctuating-risk region were significantly lower than those in the high-risk region. (2) Children in both regions were divided into four profiles based on their preschool readjustment: low-level, middle-level, upper-middle-level, and high-level groups. (3) Preschool transition practices and teachers’ turnover intention are common factors relating to preschool readjustment in both regions. Teachers’ professional development support impacted children’s preschool readjustment only in the high-risk region. The findings inform the design of targeted interventions to help children readjust to preschool across different risk regions.

## 1. Introduction

With the onset of the COVID-19 pandemic in December 2019, schools were shut down in many countries in order to reduce the contagious effects of the COVID-19 virus and to relieve the healthcare systems [1]. More than 90% of children stayed away from schools due to countries’ physical distancing policy [2]. China was at the forefront of the COVID-19 pandemic [3], and by September 2020, most preschools in China had resumed offline teaching. However, the virus continued to mutate around the world after that. China emphasizes putting people’s safety and health first, unswervingly adhering to the “dynamic zero-out” strategy [4]. Children may face ongoing interruptions in their education because of irregular rolling lockdowns and school closures under the background of normalization of pandemic prevention and control. Based on the current development of the pandemic, schools need to be prepared for possible recurring and prolonged school closures [5].

Preschool adjustment is important for children’s participation in educational activities [6,7]. Children had gradually adjusted to preschool before the outbreak of the COVID-19. However, after a long time of school closures during the pandemic, there may be maladjustment in daily routine, interaction with peers and teachers, and participation in activities when children return to preschool [8,9]. At present, little is known about young children’s preschool readjustment following school closures. The timing and severity of the COVID-19 outbreak varied from place to place. In China, researchers divided the country into risk regions based on the timing and severity of the outbreak. Risk regions had different response strategies and quarantine times, and thus, children’s preschool readjustment may also differ by region. This study aimed to understand children’s preschool readjustment and the factors influencing readjustment in different risk regions.

## 2. Literature Review

### 2.1. Children’s Preschool Readjustment following Long-Term School Closure

The COVID-19 pandemic has had a widespread impact on the lives and learning of all individuals. Children are among the most susceptible populations to be affected amid the uncertainty of the situation and the imposition of lockdowns [10,11,12]. They experienced higher levels of frustration and sadness [13,14]. The lack of face-to-face contact with classmates, friends, and teachers decreased children’s social stimulation and affected the development of their social skills [15]. Along with families experiencing unexpected disruptions to their daily lives and welfare, half of caregivers reported their child being less motivated in learning since schools were closed; one-third of children cried more often since the COVID-19 pandemic began; and some were speaking less fluently or destroying things more often [16].

Preschoolers may be more prone to maladjustment when they return to preschool. Some studies reflect the lag in young children’s behavioral, social, and academic adjustment during school closures in the COVID-19 pandemic [8,9], and unlike older students, young children typically cannot participate in distance learning without assistance from a parent or other caregiver [17]. Therefore, compared with primary and middle school students, preschool children have less contact with preschool during any isolation period. In other words, the preschool children who return to preschool may be unfamiliar with the daily routines, activities, peers and teachers of the preschool, and may not adjust to the situation. The above factors make the preschool readjustment of children following school closures during the pandemic particularly worthy of attention.

Preschool adjustment includes the acquisition of academic readiness skills, behavioral regulation at school, and socially skilled interactions with teachers and peers [18,19,20,21]. This adjustment process is important for the development of young children [6,22,23]. Children who adjust effectively are more likely to be accepted by peers and given more instruction and positive teacher feedback [7,24]. Conversely, poor adjustment to preschool can set the stage for more generalized social problems [25], poor grades and school failure [26,27,28,29]. Similar to the dimensions of preschool adjustment, the readjustment period is expected to deal with several negative sequelae emerging from difficulties in adjusting to the school routine, students’ relationships with teachers and peers, and academic pressure [30]. However, there is limited research on preschool children’s readjustment.

There are differences in children’s preschool readjustment. Latent profile analysis (LPA) provides the possibility to explore the preschool readjustment of children. LPAis a person-centered approach that uses continuous variables to group cases into subgroups based on potential similarities [31,32]. This approach avoids the shortcomings of insufficient indicators in traditional cluster analysis, makes full use of all sample data to estimate the probability of individuals belonging to a specific profile, and allows for an examination of heterogeneity in profiles. Paying attention to the heterogeneity between individuals enables more accurate descriptions of the quantitative differences between individuals, and allows multi-dimensional qualitative differences between individuals to be integrated into the analysis. Therefore, we used LPA to identify children’s distinct patterns in their preschool readjustment levels.

### 2.2. Risk Regions in the COVID-19 Pandemic

The timing and severity of the COVID-19 outbreak varied from place to place. Some Chinese researchers divided the cities of China into regions based on trends in the evolution of the epidemic [33,34,35]. Hubei province was hit the earliest and hardest by the pandemic, had the largest number of confirmed COVID-19 cases and deaths throughout China [36], and was characterized as a high-risk region with the toughest measures for family confinement. After the outbreak in Hubei, many provinces entered a period of concentrated outbreaks. Young children in some provinces such as Beijing and Xinjiang experienced the first wave of the epidemic immediately after Hubei, and then further outbreaks between June and August 2020. They had to quarantine again and reduce outdoor activities before schools reopened. Thus, these areas were termed fluctuating-risk regions [34].

On 27 January 2020, the Ministry of Education issued a notice about postponing the 2020 spring semester. Schools in all regions of the country were closed, and the local education administrative department determined the date of the reopening of preschools following the unified deployment of the local party committee and government [37]. Children in both high- and fluctuating-risk regions resumed offline education in September 2020. Although preschools reopened at approximately the same time, the nature of the pandemic differed across regions. The outbreak in Hubei province was the most serious, with children facing long and strict quarantine times. However, it was relatively unaffected in terms of the re-opening of preschool. The outbreaks in Beijing and Xinjiang occurred later, and although the overall severity were lower than that in Hubei, there were repeated outbreaks occurred near the reopen of schools.

Researchers studying the development of children during COVID-19 mainly focused on those in the high-risk regions [36]. However, with the evolution of the epidemic, the transmissibility of the virus is increasing [33]. The process of intermittent school closings and re-openings may last months to years depending on distribution in different locations [5], and the fluctuating-risk regions are likely to become a typical pattern. The differences between two regions may have an impact on children’s readjustment. LPA is an appropriate way to understand the internal characteristics of different groups [38]. The current study therefore used LPA to explore the differences in children’s preschool readjustment between regions of high and of fluctuating risk.

### 2.3. Preschool Factors Related to Children’s Preschool Readjustment

School-based transition practices influence young children’s school readjustment. Studies analyzing the influence of school transition practices are often based on the ecological and dynamic model of transition [39]. This model suggests that positive relationships—especially between children, teachers, and parents—are essential to facilitate a smooth transition for children. Empirical studies found that family–teacher communication and coherence between home and school learning simplify children’s school adjustment and improve their school achievements [40,41]. Schulting et al. (2005) [42] summed the number of school-based transition practices reported by teachers into a total score, finding that a greater number of transition practices (such as telephone or sending of information, parent orientation at school, shortened school days, etc.) were associated with heightened academic achievement scores among children at the end of the school year. Relevant studies during COVID-19 also showed that teachers sharing learning resources with parents, supporting parents to help children with psychological counseling [5], changing the daily routines and schedules to make it more appropriate [8], and organizing activities to let children understand the epidemic situation and get familiar with the school environment [43] are conducive transition practices to children’s readjustment. Researchers and educators have increasingly emphasized the importance of school transition practices during the COVID-19 pandemic [8,9], implying that such practices may reduce parental stress and improve their childcare competence [44]. School transition practices may help young children make up for the lag in behavioral, social, and academic adjustment caused by school closures, confinement, and lack of peer interaction [2]. However, there is limited empirical evidence to support the effectiveness of these measures in easing children’s preschool readjustment across risk regions.

In addition to preschool transition practices, studies have shown that early childhood development is highly susceptible to teacher-specific factors, including teacher quality and stability [45,46]. Evidence points to the effectiveness of professional development support in improving early education quality and child outcomes [47,48,49]. The shift to remote learning in 2020 was rapid, and the challenges faced by teachers were immense. Teachers moved from providing in-classroom instruction to relying on families to support learning at home [50]. Preschool teachers are less likely to receive training and support in distance learning than their primary school counterparts [51]. Without adequate professional development support, stressors will likely build up and degrade teachers’ ability to support the whole child [52]. Moreover, research indicates that teacher turnover can interrupt the attachment between child and teacher, affecting children’s language and vocabulary skills [53], as well as their emotional stability [54]. Turnover intention is the strongest determinant of actual turnover behavior [55,56], and many teachers were found to have high levels of turnover intention during the COVID-19 pandemic [57]. Therefore, it can be inferred that teachers’ turnover intention may affect children’s preschool readjustment.

The pandemic did not impact every region equally, and the relationship between preschool factors and children’s readjustment may change accordingly. This study also investigated the relation of preschool transition practices and teacher-specific factors with children’s readjustment across risk regions.

### 2.4. The Present Study

The preschool readjustment of children after long-term school closures during the COVID-19 pandemic requires investigation. There may be some heterogeneity in children’s preschool readjustment across risk regions. Preschool transition practices and teacher-specific factors may also play important roles in children’s preschool readjustment. However, readjustment—and the factors influencing it—in different risk regions during the COVID-19 pandemic have not been well studied. This study aimed to answer the following research questions: (a) How is children’s preschool readjustment following COVID-19 pandemic-related school closures in different risk regions? (b) What profiles could be identified among children regarding their preschool readjustment in different risk regions? (c) How do preschool transition practices and teacher-specific factors contribute to children’s preschool readjustment profiles in different risk regions?

## 3. Methods

### 3.1. Participants

The epidemic regions can be divided into high- and fluctuating-risk regions according to the risk characteristics of the epidemic. Based on existing research [34], the high risk regions include Hubei Province, and the fluctuating-risk regions include Beijing and Xinjiang Province. Although Beijing and Xinjiang belong to two provinces, they are classified as the same type by epidemic region. With reference to the above classification method for sampling, a total of 2407 teachers participated in our survey, including 1008 (41.9%) in the high risk region (Hubei) and 1399 (58.1%) in the fluctuating-risk region (Beijing and Xinjiang). As shown in Table 1, teachers were evenly distributed between junior (34.6% in the high-risk region and 38.6% in the fluctuating-risk region), middle (32.5% and 33.3%, respectively), and senior (32.9% and 28.1%, respectively) classes. 56.7% and 80.0% teachers worked in public preschools in the two regions of high- and fluctuating-risk respectively. In both regions, most teachers were female (98.1% and 92.0%, respectively), with less than ten years of experience (67.6% and 87.8%, respectively) and nearly half the teachers held a junior college degree (46.5% and 49.4%, respectively).

### 3.2. Measures

Preschool teachers completed three measures: a Demographic Questionnaire, an Educational Experience Questionnaire, and a Children’s Preschool Readjustment Scale.

Demographic Questionnaire. Information about children and teachers was collected via a demographic questionnaire and included items about preschool type (1 = public preschool, 2 = private preschool); children’s grade (1 = junior, 2 = middle, 3 = senior); teacher’s gender (1 = male, 2 = female); years of experience (1 = less than 3 years, 2 = 3–5 years; 3 = 6–10 years; 4 = 11–15 years; 5 = 16 years or above); and education level (1 = junior secondary or below; 2 = senior secondary; 3 = junior college; 4 = undergraduate; 5 = postgraduate).

Educational Experience Questionnaire. The educational experience questionnaire was divided into two parts. In the first part, we identified six school transition practices during the COVID-19 pandemic by interviewing preschool teachers and referring to Schulting et al. (2005) [42]. These included: (a) preschool teachers shared learning resources with parents and understood children’s performance at home; (b) parents were supported to help children with psychological counseling before school reopened; (c) daily routines and schedules were adjusted at the beginning of preschool resumption; (d) activities were undertaken retrospectively to help children become familiar with teachers and peers; (e) epidemic-related educational activities; and (f) other transition activities were carried out. Preschool teachers were asked to identify which transition practices were implemented at their school during the COVID-19 pandemic. We computed a score for each teacher by totaling the number of endorsed transition practices. Cronbach’s alpha coefficients indicated moderate internal consistency on this index (α = 0.50). This level of internal consistency is adequate for an index of this kind because implementing all six transition practices by one teacher is unlikely [42]. In the second part, teachers were surveyed about how they experienced this process, including professional development support and turnover intention. These variables were scored using 0 and 1, with 0 = “it did not happen during the COVID-19 pandemic”, and 1 = “it did happen during the COVID-19 pandemic”. Teacher professional development support refers to the Chinese Professional Standards for Preschool Teachers (Trial) and the training content of the preschool teachers interviewed in our survey, including multiple options (e.g., home-preschool co-education strategies, organization skills for online activities, and psychological guidance). Turnover intention refers to the item in Scott et al. (1999) [58], i.e., “I seriously intend to look for another job within the next year”.

Children’s Preschool Readjustment Scale. Children’s preschool readjustment was adapted from the School Adjustment Questionnaire [59] to be more suitable for evaluating young children’s transition from school closures to preschool during the pandemic. The children’s preschool readjustment scale consisted of three dimensions and 11 items: behavioral readjustment (i.e., “Children can quickly adapt to the daily routine of preschool”, 3 items), social adjustment (i.e., “Children get along well with their peers after returning to preschool”, 4 items), and academic readjustment (i.e., “Children have age-appropriate language and communication skills”, 4 items). Teachers responded to the items on a 5-point Likert scale ranging from (1) very few to (5) nearly all. Higher scores indicated greater proportions of well-adjusted children in the class. Cronbach’s α of the subscales for behavioral readjustment, social readjustment, and academic readjustment were 0.95, 0.92, and 0.96, respectively.

### 3.3. Procedures

The study was carried out following ethical standards for the treatment of human participants. Using Wenjuanxing, a leading electronic questionnaire collection platform in China, we sent e-questionnaires to preschool teachers. The questionnaires started with an introduction: preschool teachers had the right to choose whether to participate in the research after receiving information about the research objectives and being assured that the information collected would be used solely for research purposes. The main body of the survey comprised the three questionnaires: the demographic questionnaire, educational experience questionnaire, and children’s preschool readjustment scale. The available period of the e-questionnaires was set for two weeks after schools reopened. Preschool teachers could fill out the e-questionnaires via smartphone or computer at any time in the two weeks. If they were interrupted during the survey, they could complete the questionnaire at their own convenience. After two weeks, 2434 e-questionnaires were completed and returned. Twenty-seven questionnaires were deleted for the following reasons: (1) participants completed the questionnaire in less than 3 min or (2) over 90% of responses to scaled questions were identical. From the 2407 valid questionnaires, we identified 1008 teachers in the high-risk region and 1399 teachers in the fluctuating-risk region.

### 3.4. Data Analysis

Descriptive analyses were undertaken first for the analytic sample. LPA was then used to identify the latent profiles using Mplus 7.4 [60]. LPA is a person-centered approach that uses continuous variables to divide cases into subgroups based on potential similarities [61]. We followed Nylund et al.’s (2007) [62] process for determining an optimal model that starts from a two-profile model and gradually increases the number of models while assessing the estimated fit. A range of model fit indices was considered to determine the optimal number of profiles. The main indices used included the Akaike information criterion (AIC), Bayesian information criterion (BIC), and sample-size-adjusted BIC (SSA-BIC). Lower values indicated better model fit [62,63]. Entropy is often used as an index to reflect classification accuracy, with higher values indicating better classification quality [64]. The Lo–Mendell–Rubin (LMR) Likelihood Ratio Test is significant in making a discriminant analysis to choose between two models—k classes or k − 1 classes [65]. Statistically significant *p*-values obtained from the LMR likelihood ratio test indicated that a k model was better than a k − 1 model [62]. After profiles were identified, we used SPSS 25.0 to conduct one-way analyses of variance (ANOVA) to determine whether there were differences in the three dimensions of the children’s preschool readjustment. Next, multivariate logistic regression was performed to test whether certain preschool factors were associated with the likelihood of a child having a specific feature compared with the reference group [66,67].

## 4. Results

### 4.1. Children’s Preschool Readjustment in the COVID-19 Pandemic

According to the teacher’s report, the means of children’s preschool readjustment was 3.74 (SD = 0.92). More than one third (38.8%) of Chinese teachers reported that the child did not readjust well to preschool when he or she came back from home in the COVID-19 pandemic. Among them, teachers evaluated the children’s social readjustment more positively. A total of 69.3% of teachers reported that children like to go to preschool, and 64% of teachers reflected that children get along well with other children after returning to preschool during the pandemic. At the same time, teachers’ evaluation on children’s academic readjustment was more negative. In this case, 41.0% teachers thought that children didn’t pay attention after returning to school.

Using independent sample *t*-tests, we found significant differences in children’s preschool readjustment between high- and fluctuating-risk regions. In the fluctuating-risk region, the scores of children’s total preschool readjustment (*t* = 17.195, *p* < 0.001) and dimensions of behavioral readjustment (*t* = 16.273, *p* < 0.001), social readjustment (*t* = 14.736, *p* < 0.001), and academic readjustment (*t* = 17.519, *p* < 0.001) were significantly lower than those in the high-risk region (see Table 2 for details).

### 4.2. Latent Profiles of Children’s Preschool Readjustment by Risk Region

The LPA procedure was performed with preschool children in high- and fluctuating-risk regions to identify groups of children with similar preschool readjustment properties. Table 3 presents the LPA model fit indicators from the two- to five-profile models of young children in high- (*n* = 1008) and fluctuating- risk (*n* = 1399) regions. The entropy values of the four models in this study all exceeded 0.80. Based on AIC, BIC, and SSA-BIC values, the five-profile model showed lower indicators in the samples from high- and fluctuating-risk regions. However, the LMR of the five-profile model in the high-risk region sample was not significant, indicating that the five-profile was not optimal compared with the four-profile model. Although the five-profile LMR in the fluctuating-risk region was significant, one of the five profiles accounted for 3%—lower than the 5% cut-off—making it difficult to confidently represent a distinct grouping that might be generalizable to other samples [68]. Similarly, the five-profile model in the fluctuating-risk region sample did not show an optimal model fit. Taking all indicators together, we selected the four-profile model as the optimal model for children in both regions.

ANOVA was conducted with post-hoc tests to examine whether significant differences existed across children’s preschool readjustment within each profile in the two regions (Table 4). Tukey’s honest significant different post hoc tests confirmed that the four profiles differ significantly for each pair regardless of risk region. The distribution of scores for children’s preschool readjustment was generally consistent in the high- and fluctuating-risk regions, which did not intersect in the three dimensions of preschool readjustment. Therefore, the profiles of the two regions were named in the same way and are described further in the following sections.

Profile 1: Low-Level Group. In the first profile, the children’s preschool readjustment scores were less than or equal to 2.19 in both regions. They were characterized by the lowest performance in behavioral, social, and academic readjustment, and named accordingly as the low-level group. This group included 5% (*n* = 50) of children in the high-risk region and 9% (*n* = 124) of children in the fluctuating-risk region.

Profile 2: Middle-Level Group. Children’s scores for preschool readjustment ranged from 2.89 to 3.25 in the second profile in both regions. These scores were significantly higher than those in the low-level group but significantly lower than those in other groups, representing moderate behavioral, social, and academic readjustment performance. This profile was therefore called the middle-level group. The proportion of children in the middle-level group from the fluctuating-risk region (34%, *n* = 481) was higher than those from the high-risk region (18%, *n* = 183).

Profile 3: Upper-Middle Level Group. The third profile was characterized by preschool readjustment scores for the three dimensions that ranged from 3.65 to 4.06 in both regions. These scores were significantly higher than those in the low-and middle-level groups but significantly lower than the fourth group. Children with this profile were designated as the upper-middle level group. The group included 36% (*n* = 361) of children in the high-risk region and 33% (*n* = 456) of children in the fluctuating-risk region.

**Figure 1:**
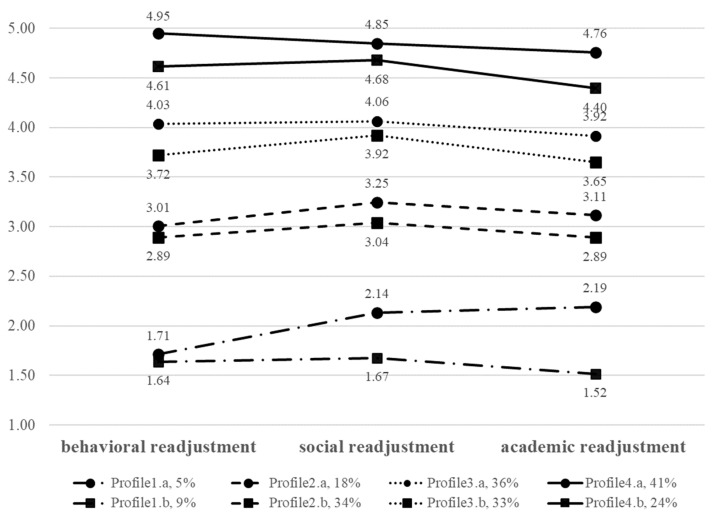
The four profiles of children’s preschool readjustment in different risk regions. Note: a = high-risk region, b = fluctuating-risk region.

Profile 4: High-Level Group. In the fourth profile, children’s preschool readjustment scores for the three dimensions ranged from 4.40 to 4.95 in both regions, demonstrating excellent performance in behavioral, social, and academic readjustment. This profile was named as the high-level group. The proportion of children in the high-level group from the fluctuating-risk region (24%, *n* = 338) were lower than those from the high-risk region (41%, *n* = 414). Figure 1 provides an overview of the four profiles.

### 4.3. Preschool Factors Associated with the Profiles of Preschool Readjustment by Risk Region

Teachers reported their educational experiences during COVID-19: in high- and fluctuating-risk regions, respectively, 83.5% and 79.1% of teachers, shared learning resources with parents and understood children’s performance at home; 95.4% and 93.4% of teachers supported parents to help children with psychological counseling before schools reopened; 24.1% and 32.5% of teachers adjusted daily routines and schedules when children returned to preschool; 71.4% and 70.7% of teachers carried out activities to help children become familiar with teachers and peers; and 87.4% and 86.0% of teachers organized epidemic-related educational activities. In addition, in the high- and fluctuating-risk regions, respectively, 71.6% and 54.3% of teachers received comprehensive professional development support; and 18.7% and 15.1% of teachers had higher turnover intention during COVID-19. The influence of preschool transition practices and teacher-specific factors on the classification of profiles in different risk regions during the COVID-19 pandemic were examined using multivariate logistic regression. Children’s grade and preschool type were used as control variables; membership of one of the four latent profiles as the dependent variable; and the high-level group was used as the reference group for the other three groups.

As shown in Table 5, the multivariate logistic regression indicated that preschool transition practices and teacher turnover intention were the common factors significantly relating to children’s membership of preschool readjustment profiles in both regions. However, these two factors present the opposite relation. Preschool transition practices increased the likelihood of a child being classified in the high-level group. More precisely, preschool transition practices were statistically significant factors for the middle-level vs. high-level group (OR = 0.814, *p* < 0.05) in the high-risk region, and in the low-level vs. high-level group (OR = 0.724, *p* < 0.001), middle-level vs. high-level group (OR = 0.683, *p* < 0.001), and upper-middle-level vs. high-level group (OR = 0.819, *p* < 0.01) in the fluctuating-risk region. In contrast, turnover intention decreased the odds of membership and was a statistically significant factor for the low-level vs. high-level groups (high-risk: OR = 2.426, *p* < 0.05; fluctuating-risk: OR = 2.176, *p* < 0.01), middle-level vs. high-level groups (high-risk: OR = 3.069, *p* < 0.001; fluctuating-risk: OR = 1.565, *p* < 0.05). In addition, teachers’ professional development support was related to children’s preschool readjustment in the high-risk region exclusively, increasing the likelihood of children being classified in the high-level group. Professional development support was a significant factor for the upper-middle-level group vs. high-level group (OR = 0.948, *p* < 0.05) in the high-risk region.

## 5. Discussion

The COVID-19 pandemic and measures to mitigate its spread affected every facet of education and society [69,70]. During the 2020 spring semester, most preschools in China were temporarily closed. These changes in daily life and the external environment made preschool readjustment difficult for some children. The current study showed that the overall preschool readjustment of children in the high- and fluctuating-risk regions was at a medium level. Children’s preschool readjustment in the fluctuating-risk region lagged behind that in the high-risk region. Four profiles of preschool readjustment were identified in the two regions. Furthermore, transition practices and turnover intention were common factors influencing children’s preschool readjustment in the two regions. In contrast, teachers’ professional development support was associated with children’s readjustment in the high-risk region only. These findings are discussed in more detail below.

### 5.1. Overall Preschool Readjustment Status of Children in the COVID-19 Pandemic

Preschool readjustment involves children’s behavioral regulation at school, socially skilled interactions with teachers and peers, and acquisition of academic readiness skills [19,21]. We found that children’s overall preschool readjustment was at a medium level after nearly half a year of school suspension. On the one hand, these results reflect that children’s readjustment is worthy of attention under the context of repeated and uncertain epidemics [17]. Because of the sudden onset of the pandemic, neither preschool teachers nor parents were prepared to help children make the transition to preschool. It may also have been the first time the children themselves experienced such a long interruption in their education. The severity and instability of the pandemic has taken a toll on children, increasing their chances of poor preschool adaptation. Therefore, some children have maladaptive problems. On the other hand, the reassuring result of medium level reflects the efforts of the Chinese government and preschools. During school closures, the Ministry of Education regarded scientific planning and arrangements for the 2020 autumn semester as the most important tasks across the country’s education system [71]. Preschools were guided regarding strict healthcare, adherence to games as the basic activities, and the careful structuring of children’s daily life [71]. These initiatives were beneficial in preparing children for preschool readjustment.

Children differed in their preschool readjustment across risk regions. Results indicated that the behavioral, social, and academic readjustment of children in the fluctuating-risk region were less optimal than in the high-risk region. Two reasons may explain this inconsistency. Children in the high-risk region generally received more attention during the pandemic [72,73], and children did not experience another outbreak for three to four months before school reopened. By contrast, the end dates of the concentrated outbreak in the fluctuating-risk region were closer to the start of the autumn semester in 2020. Affected by the repeated fluctuations and sudden outbreaks of the epidemic, preschools may have paid more attention to COVID-19 prevention and control as they prepared to reopen schools, and children’s readjustment was relatively neglected.

### 5.2. Latent Profiles of Children’s Preschool Readjustment

The types of children’s preschool readjustment in high- and fluctuating-risk regions was consistent. That is, children in both regions could be divided into four profiles. These profiles did not intersect in the three dimensions of preschool readjustment, possibly because of the high correlation between children’s social, academic, and behavioral development [74,75]. Consistent with the descriptive analysis results, children’s overall preschool readjustment was at a medium level, and preschool readjustment for children in the fluctuating-risk region were less optimal than in the high-risk region (i.e., 18%/34% in the middle-level group; 36%/33% in the upper-middle-level group).

LPA provided a more nuanced understanding of variations in children’s preschool readjustment. The study indicated that a small number were classified into the low-level group (5% in the high-risk region and 9% in the fluctuating-risk region, respectively). Research has shown that some children regressed in development (e.g., in their speech, language, and communication skills; physical development; resilience; independence; and social and emotional difficulties) because of absence from school during the COVID-19 pandemic [8,9]. Our study confirmed these findings, indicating that the pandemic has affected children differently. Factors influencing classification during COVID-19 pandemic should be identified to ensure children are more likely to be in the high-level group.

### 5.3. Preschool Factors Related to Children’s Preschool Readjustment Profiles

Preschool transition practices and teachers’ turnover intention significantly impacted children’s preschool readjustment during the COVID-19 pandemic, regardless of whether they were in high- or fluctuating-risk regions. First, preschool transition practices potentially diminished adverse consequences of the pandemic-related school closures for children’s preschool readjustment in both regions. Transition practices in the pandemic can be roughly divided into those affecting children indirectly through their parents and those impacting children directly. Previous findings have shown that parent–teacher relationships and parent involvement are important factors in children’s social skills [76], academic achievements [77] and behaviors [78]. In the extended COVID-19 lockdown, parent–teacher communication and cooperation ensured teachers could share learning resources via online social media, e.g., procedures for establishing and maintaining predictable daily routines and guidance about parent–child interactions [79,80,81,82,83,84]. Preschool practices may also be associated with preschool readjustment for children, such as initially shortened school days, setting more flexible daily routines [85], paying more attention to young children’s socio-emotional state [8], and clarifying and helping children understand previous natural disasters [86]. Our findings showed that more comprehensive preschool practices meant better readjustment of children to preschool. Second, studies suggest that the loss of a teacher with whom a child has established a trusting relationship can affect children’s feelings of security and the development of academic and social skills [87,88]. Teachers’ turnover intention is the strongest determinant of actual turnover behavior [55], which has also been found to adversely relate to children’s preschool readjustment, probably because teachers who intended to leave had reduced energy and motivation for their work [56]. During the COVID-19 pandemic, many teachers were affected by the heavier workload and lack of guaranteed salary, which led to high levels of turnover intention [57]. The treatment of teachers during the pandemic requires further consideration to reduce the adverse effects of teacher turnover on preschool readjustment.

The Chinese education policy clearly points out that, depending on the national training program and provincial training program, the online special training of distance learning should be organized in a timely manner [70]. Schools also actively innovated training methods and content for teachers to enhance their resilience and competence during COVID-19. However, the current study showed that professional development support only influenced children in the high-risk region. Differences in teachers’ characteristics across risk regions during the pandemic may explain this inconsistent influence. The COVID-19 pandemic started earlier in the high-risk region, where teachers faced additional burdens of remote learning, supporting families experiencing hardship, and the professional demands caused by the rapidly changing impacts of the pandemic. It was difficult for preschool teachers in the high-risk region to maintain positive work conditions without support, and mounting stressors were likely to reduce teachers’ ability to support the whole child [52]. In contrast, outbreaks in the fluctuating-risk region occurred later. With gradual improvement in prevention and control mechanisms, the epidemic continued for a shorter period in these places. At the same time, teachers in the fluctuating-risk region could refer to the beneficial practices in high-risk regions to support young children’s development, making professional development support in the fluctuating-risk region both less significant and less urgent than that in the high-risk region.

## 6. Implications

At present, viruses keep mutating and the global COVID-19 pandemic is not yet over. Once the local epidemic reaches a certain level of severity, Chinese preschools need to stop offline education. In other words, preschools may face long periods of absence from school. Given this uncertain background, children may still repeatedly face the situation of school suspension and subsequent readjustment. Our results have important implications for governments and preschools.

The differences between profiles across risk regions suggest that we should pay attention to the development of children in high-risk and fluctuating-risk regions. If schools need to close suddenly—when they were meant to reopen—children may face particular challenges in readjusting to preschool. The factors influencing the two risk regions suggest that targeted intervention is needed to improve children’s preschool readjustment. This study showed that teachers’ professional development support only related to children’s preschool readjustment in the high-risk region. Offline education suddenly shifted to online education during the COVID-19 pandemic, leading to teachers in the high-risk regions needing professional development support, including home–preschool co-education strategies, organization skills for online activities, and psychological guidance. Administrators and preschool managers should devote special attention to understanding the professional development needs of teachers in high-risk regions and support them accordingly, in order to facilitate young children’s optimal readjustment to preschool.

This study found that two preschool factors are associated with children’s readjustment after school closures during the pandemic regardless of risk regions. On the one hand, preschool transition practices are positive factors for readjustment, reminding preschools to attach importance to transition practices. For example, teachers should be concerned about the status of young children at home and provide parents with rich home learning activities [80,81,82]. When children returned to school, preschools then readjusted their schedules and conducted activities in a retrospective way based on what the children had missed. They also carried out epidemic-related educational activities [42,85,86]. On the other hand, teachers’ turnover intention is a negative factor for children’s preschool readjustment. Preschool teachers experienced great work pressure during the pandemic, and their salaries were often reduced, especially in private preschools [57], leading to an increase in teachers’ turnover intention that found to be associated with children’s preschool readjustment. Preschools may lack sufficient funds to ensure smooth operation amid crises. In such instances, governments are responsible for increasing financial investment in preschools and guaranteeing the salaries of teachers [89].

## 7. Limitations

Some limitations of the present study and related directions for future research should be noted. First, social distancing policies during the COVID-19 pandemic meant only teachers’ self-reported questionnaires were collected in the current study. It was, thus, difficult to objectively understand children’s preschool readjustment and explore the deeper reasons behind the data. Second, previous studies have shown that socioeconomic status (SES) is related to children’s adjustment [90]. This study mainly explored the influences of preschool transition practices and teacher-specific factors on children’s preschool readjustment and did not fully consider the familial influences. Third, the study was cross-sectional in design, precluding us from making any strong causal inferences [84]. Therefore, future research with triangulation of methods (e.g., observation and interviews), comprehensive influencing factors, and longitudinal designs is pressing.

## 8. Conclusions

This study examined children’s preschool readjustment in high- and fluctuating-risk regions of China. We found that children’s overall preschool readjustment was at a medium level after nearly half a year of school suspension. However, children’s preschool readjustment scores in the fluctuating-risk region were significantly lower than those in the high-risk region. Children in both regions were divided into four profiles based on their preschool readjustment: low-level, middle-level, upper-middle-level, and high-level groups. Preschool transition practices and teachers’ turnover intention are common factors relating to preschool readjustment in both regions. Teachers’ professional development support impacted children’s preschool readjustment only in the high-risk region. The findings inform the design of targeted interventions to help children readjust to preschool across different risk regions.

## Figures and Tables

**Table 1:** The characteristics of the sample.

	High-Risk Region (*n* = 1008)		Fluctuating-Risk Region (*n* = 1399)	
	N	%	N	%
**Preschool type**				
Public school	572	56.7%	1119	80.0%
Private school	436	43.3%	280	20.0%
**Grade**				
Junior	349	34.6%	540	38.6%
Middle	328	32.5%	466	33.3%
Senior	331	32.9%	393	28.1%
**Gender**				
Male	19	1.9%	112	8.0%
Female	989	98.1%	1287	92.0%
**Years of experience**				
Less than 3 years	258	25.6%	488	34.9%
3–5 years	204	20.2%	403	28.8%
6–10 years	220	21.8%	337	24.1%
11–15 years	123	12.2%	87	6.2%
16 years or above	203	20.2%	84	6.0%
**Education backgrounds**				
Junior secondary or below	19	1.9%	8	0.6%
Senior secondary	162	16.1%	96	6.9%
Junior college	469	46.5%	691	49.4%
Undergraduate	354	35.1%	585	41.8%
Postgraduate	4	0.4%	19	1.3%

**Table 2:** An examination of differences in preschool readjustment in different risk regions.

Variables	High-Risk Region (*n* = 1008)	Fluctuating-Risk Region (*n* = 1399)	*t*
Behavioral readjustment	4.11 ± 0.92	3.47 ± 0.99	16.273 ***
Social readjustment	4.14 ± 0.85	3.60 ± 0.94	14.736 ***
Academic readjustment	4.03 ± 0.86	3.38 ± 0.95	17.519 ***
Preschool readjustment	4.09 ± 0.83	3.49 ± 0.89	17.195 ***

Note. *** *p* < 0.001 (two-tailed).

**Table 3:** Model fit indicators of the latent profile of children’s preschool readjustment in different risk regions.

Model	AIC	BIC	SSA-BIC	Entropy	LMRT (*p*)	Latent Profile Proportions
High-risk region (*n* = 1008)						
2	6108.54	6157.64	6125.93	0.92	1638.31 ***	0.26, 0.74
3	5214.76	5283.58	5239.11	0.92	870.32 ***	0.18, 0.39, 0.43
4	4490.65	4579.13	4521.96	0.97	706.57 ***	0.05, 0.18, 0.36, 0.41
5	4343.64	4451.79	4381.91	0.97	149.60	0.05, 0.18, 0.01, 0.36, 0.40
Fluctuating-risk region (*n* = 1399)						
2	9938.81	9991.25	9959.48	0.81	1593.54 ***	0.44, 0.56
3	8675.43	8748.84	8704.37	0.90	1228.97 ***	0.10, 0.42, 0.48
4	8359.47	8453.86	8396.68	0.85	313.15 ***	0.09, 0.24, 0.34, 0.33
5	8178.17	8293.53	8223.64	0.87	182.99 ***	0.32, 0.07, 0.03, 0.24, 0.34

Note. *** *p* < 0.001; AIC = Akaike Information Criterion; BIC = Bayesian Information Criterion; SSA-BIC = Sample-Size-Adjusted BIC; LMRT = Lo–Mendell–Rubin Test.

**Table 4:** Comparison of preschool readjustment among young children with different latent profiles by risk region.

		Behavioral Readjustment	Social Readjustment	Academic Readjustment
Low-level group (1)	a	1.71 ± 0.45	2.14 ± 0.77	2.19 ± 0.85
	b	1.64 ± 0.51	1.67 ± 0.51	1.52 ± 0.50
Middle-level group (2)	a	3.01 ± 0.25	3.25 ± 0.53	3.11 ± 0.43
	b	2.89 ± 0.49	3.04 ± 0.35	2.89 ± 0.48
Upper-middle-level group (3)	a	4.03 ± 0.22	4.06 ± 0.40	3.92 ± 0.45
	b	3.72 ± 0.53	3.92 ± 0.33	3.65 ± 0.52
High-level group (4)	a	4.95 ± 0.14	4.85 ± 0.27	4.76 ± 0.38
	b	4.61 ± 0.40	4.68 ± 0.30	4.40 ± 0.48
F	a	5574.15 ***	1102.17 ***	915.77 ***
	b	1476.00 ***	2844.28 ***	1263.74 ***
Group differences	a	1 < 2 < 3 < 4	1 < 2 < 3 < 4	1 < 2 < 3 < 4
	b	1 < 2 < 3 < 4	1 < 2 < 3 < 4	1 < 2 < 3 < 4

Note. *** *p* < 0.001; reference group: high-level group; a = high-risk region, b = fluctuating risk region.

**Table 5:** Summary of multivariate logistic regression on the influence of preschool factors on children’s preschool readjustment profiles by risk region.

		P1 vs. P4		P2 vs. P4		P3 vs. P4	
		B (SE)	OR	B (SE)	OR	B (SE)	OR
Control variables							
Preschool transition practices							
	a	−0.126 (0.152)	0.882	−0.206 (0.092)	0.814 *	−0.139 (0.075)	0.870
	b	−0.323 (0.096)	0.724 ***	−0.382 (0.067)	0.683 ***	−0.200 (0.067)	0.819 **
Teacher-specific factors							
Professional development support	a	−0.010 (0.055)	0.990	0.005 (0.034)	1.005	−0.053 (0.027)	0.948 *
	b	0.012 (0.038)	1.012	0.003 (0.025)	1.003	−0.009 (0.025)	0.991
Turnover intention	a	0.886 (0.365)	2.426 *	1.121 (0.229)	3.069 ***	0.400 (0.209)	1.491
	b	0.777 (0.284)	2.176 **	0.448 (0.213)	1.565 *	0.088 (0.222)	1.092

Note: P1 = low-level group, P2 = middle-level group, P3 = upper-middle-level group, P4 = high-level group; a = high-risk region, b = fluctuating-risk region; * *p* < 0.05, ** *p* < 0.01, *** *p* < 0.001.
